# Letter to the editor in response to “Management of a challenging open urethral defect in a patient with Fournier's gangrene: A case report” Int. J. Surg. Case Rep.

**DOI:** 10.1016/j.ijscr.2025.111922

**Published:** 2025-09-09

**Authors:** Radhika Salpekar, Vikram Satav, Radhika Salpekar

**Affiliations:** Department of Urology, Dr. D. Y. Patil Medical College and Hospital, Pimpri, Pune, Maharashtra, 411018, India

Dear Editor,

I read the case report recently published in the International Journal of Surgery Case Reports on “Management of a challenging open urethral defect in a patient with Fournier's gangrene: A case report [1].” The article has comprehensively showcased the surgical challenges encountered during the reconstruction of major perineal defects with concurrent urethral involvement [1]. The authors successfully and admirably achieved a functionally and cosmetically satisfactory outcome at the end of multiple surgeries and a prolonged hospital stay.

Drawing on our own clinical experience with similar cases, we would like to highlight an important technical consideration: the early placement of a suprapubic catheter (SPC) for urinary diversion. We have found that SPC placement not only promotes wound healing but also reduces catheter-associated infection rates [2] and facilitates safe Foley removal, especially in complex scenarios.

A prolonged history of uncontrolled type 2 diabetes mellitus in such patients is concerning on two fronts: firstly, with such an extensive infected wound with a urethral defect, the risk of ascending urinary tract infection is high (urine culture positive for *E. coli*), and secondly, one is unaware of his detrusor function due to the possibility of diabetic cystopathy. We have encountered cases where, despite a urethral repair that was adequate in form and function, the patients were unable to void after urethral Foley catheter removal due to an underactive detrusor, thus necessitating emergent suprapubic urinary diversion.

SPC offers some practical advantages: it can be maintained long-term while allowing staged and/or delayed urethral repair [3], and enables a controlled transition to spontaneous voiding, once the repair has healed. It would be difficult in this situation with a urethral defect to safely replace a per-urethral catheter every few weeks until a definitive repair is made.

A standard of care we tend to follow is, to maintain the SPC during urethral healing, and blocking it when ready for per-urethral voiding trials. Once he has done so to the surgeon's satisfaction with no significant post-void residue, the SPC can be safely removed. This approach has led to fewer infections, better wound healing, and a more reliable assessment of bladder function in our practice.

Given these benefits, we recommend routine consideration of suprapubic catheterization in the perioperative management of open urethral defects associated with severe perineal infection. Its use may further improve both short- and long-term outcomes in this challenging patient population.

We hope this suggestion adds to the evolving expertise in reconstructive urology and plastic surgery for Fournier's gangrene and look forward to continued discussion on best practices.

Thank you for considering this contribution to an important topic. I look forward to your thoughts and any future discussions regarding innovations in bladder irrigation techniques.

## Author contribution

1) Dr. Vikram Satav- Study concept, writing the paper, guidance on point of technique.
2) Dr. Radhika Salpekar- Study design, paper writing, corresponding author.

## Consent

N/A as this is a Letter to the Editor.

## Ethical approval

Ethical approval not required.

## Guarantor

Dr. Radhika Salpekar.

## Research registration number

Not applicable.

## Funding

None.

## Conflict of interest statement

None.
